# Pharmacological inhibition of thioredoxin reductase increases insulin secretion and diminishes beta cell viability

**DOI:** 10.1007/s00210-020-02046-2

**Published:** 2021-01-19

**Authors:** Dennis Brüning, Kathrin Hatlapatka, Verena Lier-Glaubitz, Vincent Andermark, Stephan Scherneck, Ingo Ott, Ingo Rustenbeck

**Affiliations:** 1 Institute of Pharmacology, Toxicology and Clinical Pharmacy, Technische Universität Braunschweig, D-38106 Braunschweig, Germany; 2 Institute of Medicinal and Pharmaceutical Chemistry, Technische Universität Braunschweig, D-38106 Braunschweig, Germany

**Keywords:** Insulin secretion, Cytosolic calcium concentration, Glucose, Thioredoxin, NADPH

## Abstract

Apparently, both a decrease in beta cell function and in beta cell mass contribute to the progressive worsening of type 2 diabetes. So, it is of particular interest to define factors which are relevant for the regulation of insulin secretion and at the same time for the maintenance of beta cell mass. The NADPH-thioredoxin system has a candidate role for such a dual function. Here, we have characterized the effects of a highly specific inhibitor of thioredoxin reductase, AM12, on the viability and function of insulin-secreting MIN6 cells and isolated NMRI mouse islets. Viability was checked by MTT testing and the fluorescent live-dead assay. Apoptosis was assessed by annexin V assay. Insulin secretion of perifused islets was measured by ELISA. The cytosolic Ca^2+^ concentration was measured by the Fura technique. Acute exposure of perifused pancreatic islets to 5 μM AM12 was without significant effect on insulin secretion. Islets cultured for 24 h in 0.5 or 5 μM AM12 showed unchanged basal secretion during perifusion, but the response to 30 mM glucose was significantly enhanced by 5 μM. Twenty-four-hour exposure to 5 μM AM12 proved to be without effect on the viability of MIN6 cells, whereas longer exposure was clearly toxic. Islets were more susceptible, showing initial signs of apoptosis after 24-h exposure to 5 μM AM12. The activity of the NADPH-thioredoxin system is indispensable for beta cell viability but may have a limiting effect on glucose-induced insulin secretion.

## Introduction

Thioredoxins (TRX) are proteins that act as antioxidants by facilitating the reduction of other proteins by cysteine thiol-disulfide exchange. They are ubiquitously expressed and are essential for practical all cellular systems (Lu and Holmgren 2014). TXRs are kept in the reduced state by the flavoenzyme thioredoxin reductase (TRX-R), in a NADPH-dependent reaction. In mammalian cells, the cytosolic and mitochondrial TRX systems, in which TRX-Rs are high molecular weight selenoenzymes, control the cellular redox environment in conjunction with the glutathione-glutaredoxin (GRX) system, consisting of NADPH, glutathione reductase, GSH, and GRX (Lu and Holmgren 2014).

The role of TRX in beta cell physiology is interesting because of two perspectives. First, it may be involved in the NADPH-mediated enhancement of insulin secretion and second, its activity may be necessary to maintain beta cell viability. The hypothesis that TRX may be involved in the metabolic amplification of insulin secretion was investigated here because of the observation that NADPH is increased during glucose stimulation (Westermark et al. 2007; Ferdaoussi et al. 2015; Attie 2015). NADPH is thus a candidate coupling compound in the still incompletely understood amplifying pathway of insulin secretion (Henquin 2000), even though it is unlikely to play an exclusive role (Panten and Rustenbeck 2008).

Originally, it was thought that NADPH would exert a stimulatory effect via glutaredoxin, whereas TRX would exert an inhibitory effect (Ivarsson et al. 2005). Later, the stimulatory role of glutaredoxin was confirmed, whereas no immediate effect of TRX downregulation was seen (Reinbothe et al. 2009). However, an essential role of TRX in maintaining beta cell viability became visible by overexpression of the TRX-interacting proteins (TXNIP), which resulted in markedly increase rates of beta cell apoptosis (Chen et al. 2008). TXNIP binds to TRX and in consequence inhibits its enzymatic activity (Patwari et al. 2006). Remarkably, TXNIP gene expression in beta cells is induced by high glucose (Chen et al. 2008). So, TRX appears to be relevant for the maintenance of the functional beta cell mass.

Pharmacological tools to affect TRX function by inhibition of TRX-R were developed in the context of anticancer research (Mustacich and Powis 2000; Urig and Becker 2006; Holmgren and Lu 2010). Alkynyl(phosphan)gold-compounds inhibit the TRX-R and proved to have remarkable antitumoral properties. AM12 inhibits TrxR with an IC_50_ value of 45 nM and a more than 200-fold selectivity compared with the functionally and structurally closely related enzyme glutathione reductase (Meyer et al. 2012).

In particular, the compound AM12 proved to specifically inhibit thioredoxin reductase without exerting additional toxic effects at the relevant concentrations. It reduced cellular respiration but showed only very minor effects as a decoupling agent of oxidative phosphorylation (Meyer et al. 2012; Andermark et al. 2016). Thus, we characterized the effects of this compound on the viability of insulin-secreting cells and islets and on glucose-induced insulin secretion to assess its suitability to investigate the role of the NADPH-thioredoxin system in beta cells.

## Materials and methods

### Chemicals

**Fig. 1:**
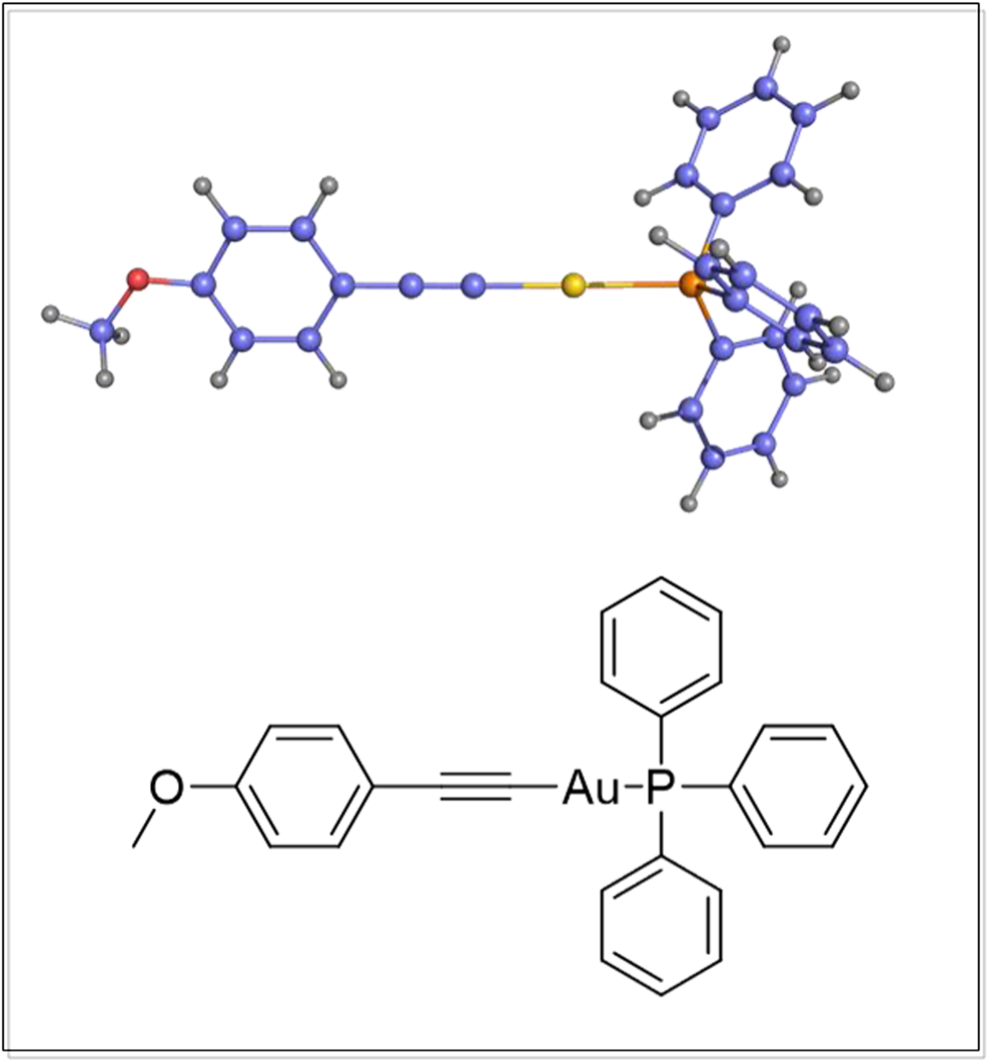
Chemical structure of the alkinyl(triphenylphosphan)gold(I)-complex AM12. [2-(4-Metoxyphenyl)ethin-1-yl](triphenyl-phosphan)gold(I)

Collagenase P for islet isolation was from Roche (Sigma, Taufkirchen, Germany). Fura-2 LeakRes (AM) was obtained from TEF-Labs (Austin, TX, USA). Cell culture medium RPMI 1640 was from Sigma and fetal calf serum (FCS Gold ADD) was from Bio & Sell (Nürnberg-Feucht, Germany). Bovine serum albumin (BSA, fraction V) and all other reagents of analytical grade were from E. Merck (Darmstadt, Germany). AM12 [2-(4-metoxyphenyl) ethyn-1-yl](triphenyl-phosphan)gold(I) was prepared as described earlier (Meyer et al. 2012; Andermark et al. 2016). The stock solution of AM12 (5 mM) was prepared in dimethylformamide (DMF) (Fig. 1).

### Islet isolation and tissue culture

Islets were isolated from the pancreas of female NMRI mice (12–16 weeks old, fed ad libitum) by a conventional collagenase digestion technique. For islet isolation and experimentation, a HEPES-buffered Krebs-Ringer medium (KR medium) was used. The composition was (mM): NaCl 118.5, KCl 4.7, CaCl_2_ 2.5, KH_2_PO_4_ 1.2, MgSO_4_ 1.2, NaHCO_3_ 20, HEPES 10, BSA 0.2% w/v. If not stated otherwise, glucose concentration was 5 mM. After collagenase digestion for 9.5 min in a shaking water bath, exchange of the KR medium, and cooling to 8 °C, the islets were collected by the use of an Eppendorf pipette under a stereomicroscope. Animal care was supervised by the regional authority (LAVES, Lower Saxony, Germany) and conformed to the current EU regulations. Insulin-secreting MIN6 cells were kindly provided by Jun-Ichi Miyazaki (Miyazaki et al. 1990) and cultured in DMEM medium (high glucose, 4.5 g/l = 25 mM, final glutamine concentration 6 mM) containing 10% FBS and penicillin/streptomycin in a humidified atmosphere of 95% air and 5% CO_2_ at 37 °C. Since the stock solution of AM12 was prepared with DMF, DMF was always present at a concentration of 0.1% (vol/vol).

### Viability and apoptosis measurements

The yellow water-soluble MTT (3-(4,5-dimethylthiazol-2-yl)-2,5-diphenyltetrazoliumbromide) is cleaved by microsomal enzymes to give a blue-violet water-insoluble MTT-Formazan, which can be measured after solubilization at 570 nm (Mosmann 1983). The concomitant reduction is dependent on the availability of NADH and NADPH (Berridge and Tan 1993). Thus, the MTT test measures primarily the metabolic activity from which viability is extrapolated (Janjic and Wollheim 1992; Berridge et al. 1996).

For this reason, viability was also checked by the live/dead assay (Papadopoulos et al. 1994). Here, the non-fluorescent membrane-permeable calcein AM ester is cleaved in the cytosol of living cells, whereby a green fluorescence is produced. Fluorescent calcein is retained in the intact cells. Ethidium homodimer III is excluded from living cells but can reach the nucleus of dead cells where it binds to nuclear DNA, whereby the red fluorescence is about 40-fold intensified. Calcein is excited at 494 nm and emits at 517 nm, ethidium homodimer at 530 nm and 620 nm, respectively. Cells were loaded with 1 μM calcein AM und 2 μM ethidium homodimer for 30 min at room temperature in the dark.

The fluorescence was measured by a Cary Eclipse fluorometer equipped with a microplate accessory (Agilent Technologies, Cary Eclipse WinFLR software, Ver. 1.2). The percentage of living cells was calculated: F(517) sample − F(517) min/F(517) max − F(517) min. Correspondingly, the percentage of dead cells was calculated: F(620)sample − F(620) min/F(620) max − F(620) min, where sample refers to cells loaded with both indicators, min to untreated cells loaded with either calcein or ethidium homodimer and max to saponin-treated cells loaded with either calcein or ethidium homodimer. Green and red fluorescent cells were also visualized by fluorescence microscopy using a Zeiss Fluar objective (10×; 0.5 N.A.). Excitation and emission were described above; dichroic separation was at 498 and 562 nm, respectively.

To assess the viability of AM12-exposed islets, spinning disk laser scanning microscopy was used. Additionally, the occurrence of early steps of apoptosis was checked by annexin V assay (ABP Biosciences, Rockwell, MD, USA). Andy Fluor 488-coupled annexin V binds to the outer leaflet of the plasma membrane if phosphatidylserine is present, which indicates the onset of apoptosis (Martin et al. 1995). The number of dead cells is again indicated by the red fluorescence of ethidium homodimer III.

After loading with the indicators, islets were placed on Petri dishes with a glass bottom (ibidi GmbH, Gräfelfing, Germany) and placed on the stage of an inverted Nikon Ti2-E microscope fitted with a Yokogawa CSU W1 spinning disk unit. The green fluorescence of calcein and of Alexa Fluor 488 was excited at 491 nm, the red fluorescence of ethidium homodimer at 561 nm, and collected by a Nikon CFI Plan Apochromat Lambda S40 XC Sil objective (40×, 1.25 N.A.), which is designed to image thick specimen of living cells. Images were acquired by a sCMOS camera (Prime BSI, Teledyne Photometrics, Tucson, AZ, USA) under control of Visiview Premier software (Visitron Systems, Munich, Germany).

### Measurement of insulin secretion

Batches of freshly isolated 50 islets were introduced into a purpose-made perifusion chamber (37 °C) and perifused at 0.9 ml/min KR medium as described above, which was saturated with 95% O_2_ and 5% CO_2_ and contained glucose as described in the “Results” section. This medium was also used for the microfluorometric measurements. The insulin content in the fractionated efflux was determined by ELISA according to the manufacturer’s protocol (Mercodia, Uppsala, Sweden).

### Measurement of the cytosolic Ca^2+^ concentration ([Ca^2+^]_i_)

Prior to the experiment, the islets were loaded with Fura-2 LeakRes/AM (2 μM in KR medium) for 45 min at 37 °C. Five islets were then inserted in a temperature-controlled perifusion chamber (36 °C) on the stage of a Zeiss Axiovert 135 microscope equipped with a Zeiss Fluar (10×, 0.5 N.A.) objective and perifused with KR medium. The fluorescence of each islet (excitation at 340 or 380 nm, dichroic separation at 400 nm, emission 510 ± 40-nm bandpass) was recorded with a cooled CCD camera (Pursuit, Diagnostics Instruments, Sterling Heights, MI, USA) and evaluated using Visiview software (Visitron, Munich, Germany).

### Statistics

Results are presented as mean ± SEM. GraphPad Prism5 software (GraphPad, LaJolla, CA) was used for statistic calculations and non-linear curve fitting (ELISA).

## Results

**Fig. 2:**
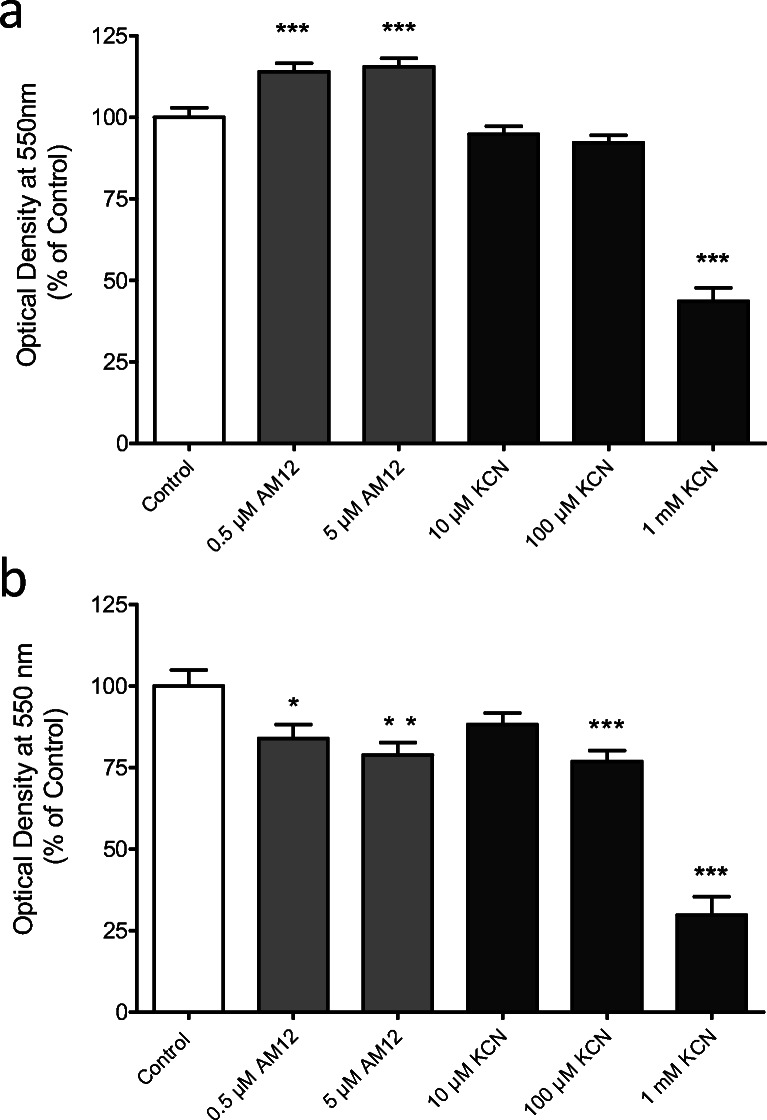
Effect of AM12 on the viability of MIN6 cells as measured by the MTT assay method. MIN6 cells were cultured for 24 h (**a**) or 48 h (**b**) in the presence of 0.5 or 5 μM AM12 or 10, 100, or 1000 μM KCN. The data are means ± SEM of 6 (**a**) or 3 (**b**) experiments. The data were normalized setting the mean value of the respective control value to 100% *p < 0.05; **p < 0.01; ***p < 0.001 (ANOVA corrected for multiple comparisons by Dunnett’s post hoc test)

Since AM12 is able to induce cell death, it was first investigated for how long insulin-producing cells need to be exposed to AM12 to show signs of diminished viability. After 24-h exposure, neither 0.5 nor 5 μM AM12 had reduced MTT conversion, rather MTT conversion was slightly but significantly increased (Figs. 2a). 100 µM KCN was slightly effective and 1 mM KCN was strongly effective to reduce MTT conversion. A 48-h exposure led to a moderate significant reduction (to about 80%) by both 0.5 and 5 μM AM12. This effect size was comparable with the effect of 100 μM KCN, whereas 1 mM KCN reduced MTT conversion to about 25% (Fig. 2b).

**Fig. 3:**
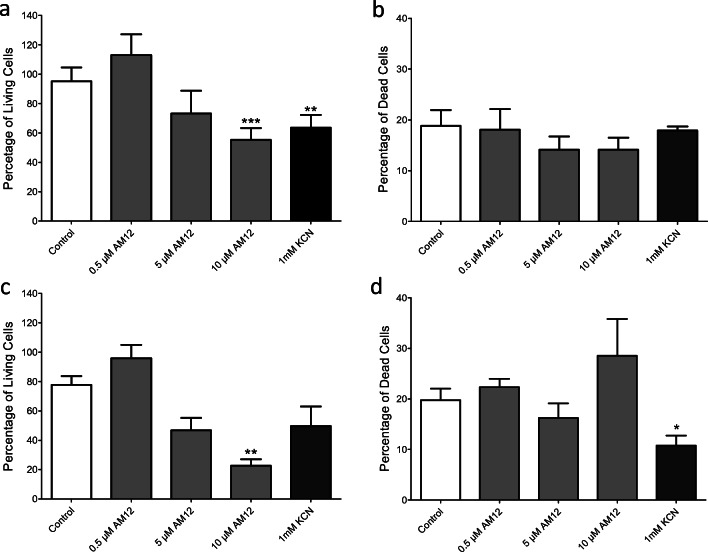
Effect of AM12 on the viability of MIN6 cells as measured by the live/dead assay method. MIN6 cells were cultured for 24 h (**a**) or 48 h (**b**) in the presence of 0.5 or 5 μM AM12 or 100 or 1000 μM KCN. The data are means ± SEM of 3 experiments each. *p < 0.05; **p < 0.01; ***p < 0.001 (ANOVA, corrected for multiple comparisons by Dunnett’s post hoc test)

The results of the MTT conversion test were checked by using the live/dead assay. Again, MIN6 cells were exposed for 24 h or 48 h to 0.5, 5, or 10 μM AM12 or 1 mM KCN. After both exposure times, there was a concentration-dependent decrease in the number of living cells; however, only 10 μM AM12 was significantly effective as compared to control. After 48 h, the concentration dependency was clearly steeper (Fig. 3a and c). Unexpectedly, the percentage of dead cells did not show a concentration-dependent increase, after 48 h of exposure to KCN, there was even a significant decrease (Fig. 3b and d). Assuming that early interference with cell proliferation had contributed to these observations, a clarification by fluorescence microscopy was sought.

**Fig. 4:**
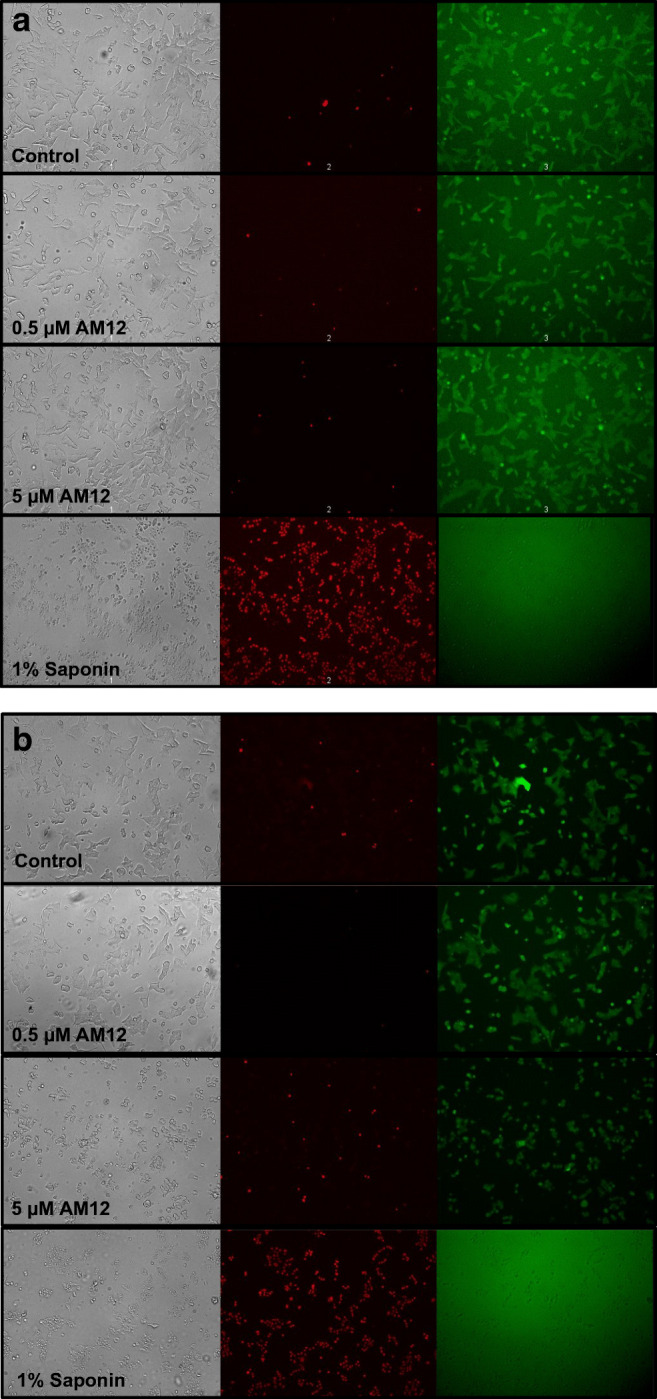
Effect of AM12 on the viability of MIN6 cells as visualized by the live/dead assay method. MIN6 cells were cultured for 24 h (**a**) or 48 h (**b**) in the presence of 0.5 or 5 μM AM12 or 1% saponin. The micrographs show the cells in transmitted light (left), with the red ethidium homodimer fluorescence (middle) and with the green calcein fluorescence (right). The micrographs show that after 24 h of AM12 exposure, the number of red fluorescent cells was not higher than after 24 h under control condition. After 48 h, there was a slightly higher number of dead cells following exposure to 5 μM but not 0.5 μM AM12. Typical observations of 3 experiments

Fluorescence microscopy confirmed that 24-h exposure to 0.5 μM or 5 μM AM12 did not increase the number of dead cells and left the metabolically active cells as well as the total cell number unchanged (Fig. 4a). Saponin, which was used as a positive control, led to a high number of red fluorescent spots which correlated with a nearly complete absence of intact cells (Fig. 4a). After 48-h exposure, 0.5 μM AM12 was still without effect on the viability, whereas 5 μM led to a moderate increase in the number of red fluorescent cells, a moderate decrease in green fluorescent cells and a fragmented appearance of an overall diminished cell number in transmitted light (Fig. 4b).

**Fig. 5:**
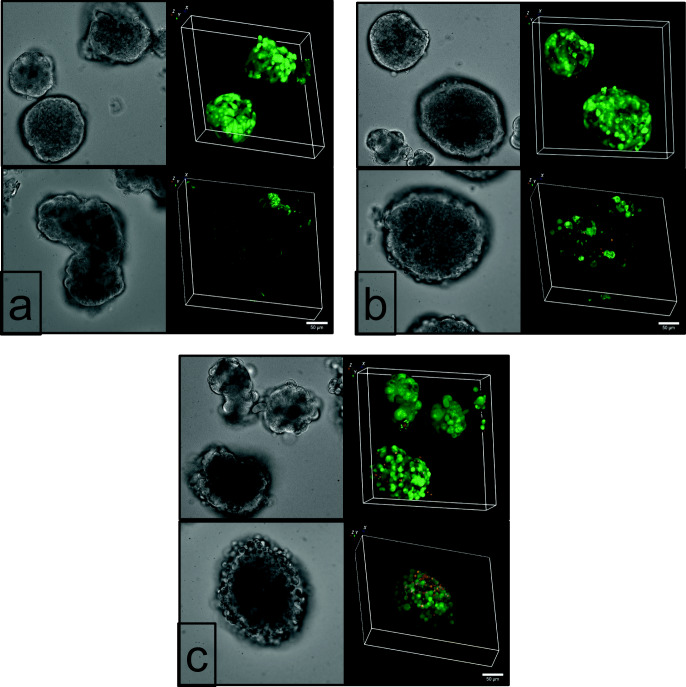
Viability and apoptosis of islets exposed to AM12 for 24 h in cell culture. Islets were cultured for 24 h in the presence of 0.1% DMF (control, **a**) or 0.5 μM AM12 (**b**) or 5.0 μM AM12 (**c**). The left micrographs show the islets in transmitted light; the right micrographs show the corresponding fluorescence. The upper right micrographs show the calcein fluorescence indicating viability in green and the ethidium homodimer fluorescence indicating cell death in red; the lower right micrographs show the annexin V fluorescence indicating initial stages of apoptosis in green and the ethidium homodimer fluorescence indicating cell death in red. Typical observations of 3 experiments each

Culturing isolated islets for 24 h in 0.5 μM AM12 did not affect the islet appearance in transmitted light and calcein fluorescence (Fig. 5a and b). Likewise, red nuclei were scarce and superficial as also occasionally visible with islets cultured in 0.1% DFM (control). The exposure to 5.0 μM AM12, however, left a clear mark in that most islet cells were rounded and in the majority of islets, the cell-cell contacts were loosened. Red nuclei, though not numerous, were regularly apparent in the islet periphery (Fig. 5c). This observation led us to check for signs of apoptosis. Islets cultured for 24 h in 0.5 μM AM12 showed occasional annexin V labeling in the outer cell layer, a feature which also, but less frequently seen with control-cultured islets (Fig. 5a and b). The 24-h exposure to 5.0 μM AM12 left again a much clearer mark in that the majority of islet cells were labeled by annexin V (Fig. 5c).

**Fig. 6:**
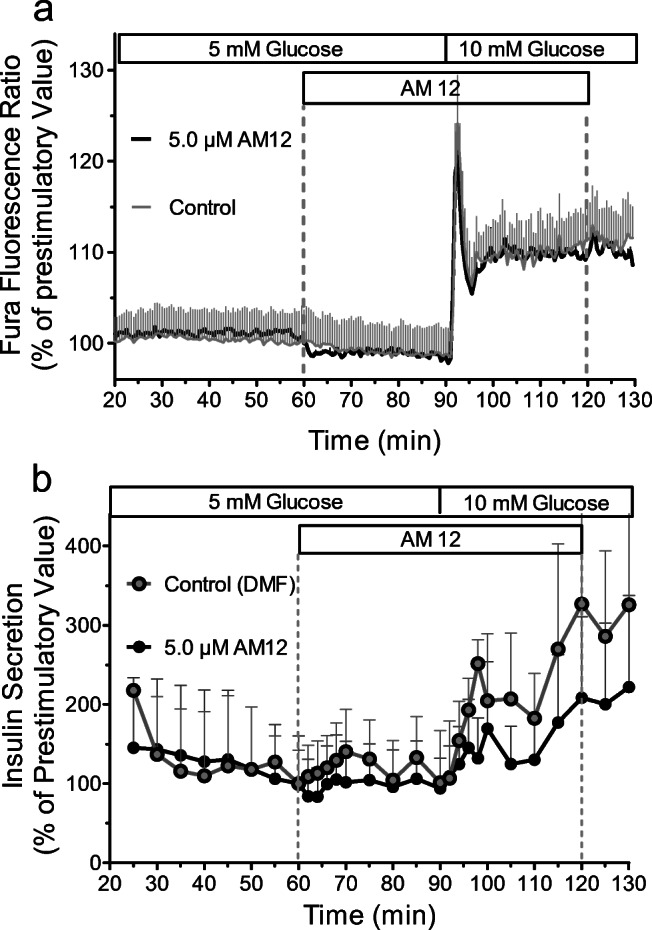
Acute effect of AM12 on the [Ca^2+^]_i_ of freshly isolated pancreatic islets (**a**) and the simultaneously measured insulin secretion (**b**). 10 freshly isolated were loaded with Fura 2/AM (leak resistant) and were perifused at 36 °C with Krebs-Ringer medium containing 5 mM glucose for 90 min; then, glucose was raised to 10 mM for another 40 min. AM12 was present from 60 to 120 min. The Fura fluorescence ratio of all islets was measured per experiment; the secretion was measured in the fractionated efflux of these islets. The secretion rate at 60 min, which was normalized to 100%, was 8.9 ± 0.9 pg/min × islet. Data are means ± SEM of 3 experiments each

The ability of AM12 to affect insulin secretion was initially tested by perifusing freshly isolated islets and simultaneously measuring [Ca^2+^]_i_ and insulin release (Fig. 6). In the presence of a basal glucose concentration (5 mM), there was no effect of 5 μM AM12. A slight, non-significant inhibitory effect was noted when glucose was raised from 5 to 10 mM. During the entire experiment, [Ca^2+^]_i_ was not affected by AM12 but responded vigorously to the increase in glucose.

**Fig. 7:**
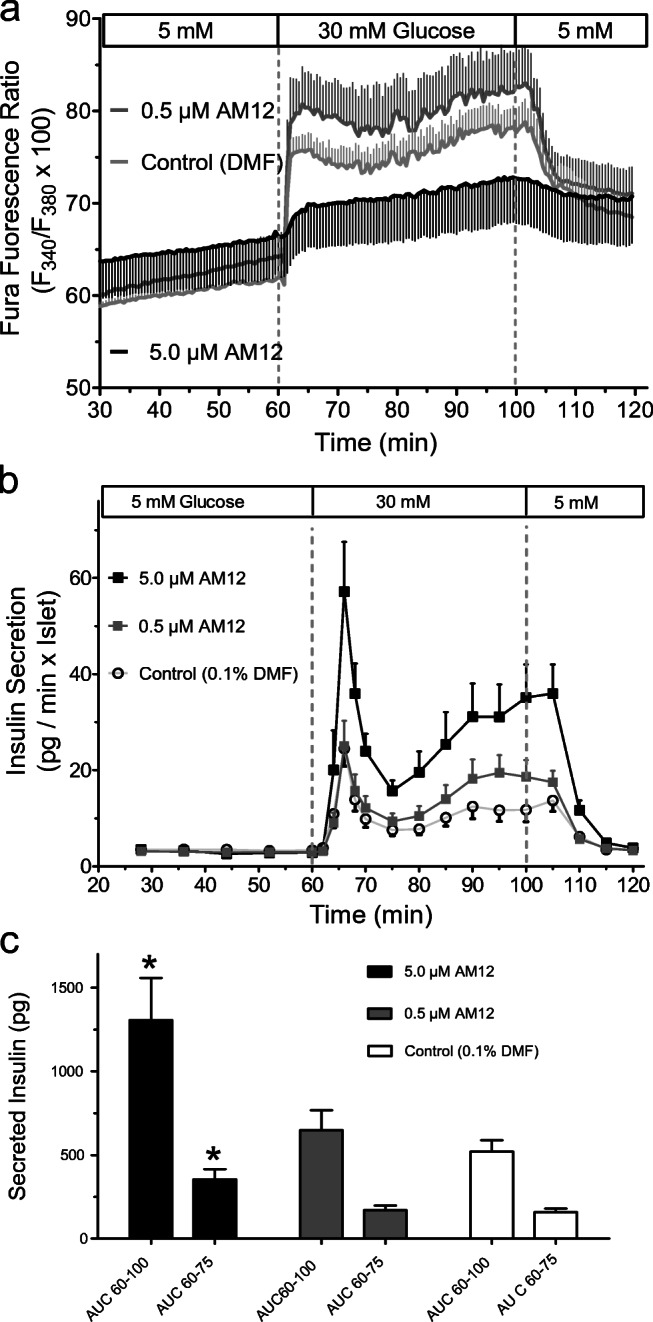
Effect of 30 mM glucose on the [Ca^2+^]_i_ (**a**) and insulin secretion (**b** and **c**) of AM12-exposed or control islets. Islets were cultured for 24 h in the presence of 0.5 or 5.0 μM AM12 or in the presence of 0.1% DMF (control). AM12-exposed islets and control-cultured islets were perifused with KR medium containing 5 mM glucose for 60 min; then glucose was raised to 30 mM for 40 min and lowered back to 5 mM for another 30 min. **a** To measure the cytosolic Ca^2+^ concentration, islets were loaded with Fura 2/AM (leak resistant) and the Fura fluorescence ratio was measured during the perifusion. Data are means ± SEM of 5 experiments each. **b** To measure insulin secretion, 50 islets were inserted in a temperature-controlled perifusion chamber and the insulin content was measured in the fractionated efflux. **c** Comparison of the insulin release is shown in **b** as the AUC between 60 and 100 min (total) and 60 and 75 min (first phase). *p < 0.05, unpaired two-tailed *t* test with Welch’s correction, as compared with control. Data are means ± SEM of 9, 5, or 8 experiments for 5 μM AM12, 0.5 μM AM12 and control, respectively

Assuming that AM12 did not reach a sufficiently high intracellular concentration during the acute exposure, its ability to affect insulin secretion was then tested by perifusing islets which had been cultured for 24 h in the presence of 0.5 or 5.0 μM AM12 or to 0.1% DFM (control). In this experiment, the glucose concentration was raised from 5 to 30 mM. In the presence of 5 mM glucose, the insulin secretion of islets cultured in the presence of either AM12 concentration was not different from the one of control-cultured islets. When the glucose concentration was raised to 30 mM, the secretion of islets cultured in the presence of 0.5 μM was practically identical to that of control-cultured islets (Fig. 7). Islets which had been cultured in the presence of 5 μM AM12 responded to 30 mM glucose with the same biphasic pattern, but the amount of released insulin was significantly higher. Upon re-exposure to basal glucose, all three treatment groups re-established prestimulatory secretion levels within 20 min.

In the presence of 5 mM glucose, the [Ca^2+^]_i_ of perifused islets was slightly higher after exposure to either concentration of AM12 than after control culture. The increase of [Ca^2+^]_i_ caused by 30 mM glucose was moderately higher in 0.5 μM AM12-cultured islets than in control-cultured islets but showed the same kinetics. Islets cultured in 5.0 μM AM12, in contrast, showed a much-reduced response to the glucose stimulus. At the end of the washout period, the [Ca^2+^]_i_ was closely similar in all three groups (Fig. 7).

## Discussion

This investigation aims at characterizing the effect of TRX-R inhibition on insulin secretion and beta cell viability. TRX-R is the only enzyme known to catalyze the reduction of TRX (Mustacich and Powis 2000) and is thus an indispensable component in the TRX system. Electrons are derived from NADPH via TRX-R and are transferred to the active site of TRX, which reduces protein disulfides. Hence, inhibition of this enzyme should inevitably inactivate TRX-dependent processes (Holmgren and Lu 2010).

The inhibition of TRX-R by AM12 may on one side lead to a less reduced state of TRX and of TRX-dependent proteins. On the other hand, it may lead to increased levels of NADPH as long as the beta cell metabolism and in consequence NADPH generation is not affected by diminished levels of reduced TRX. Indirect evidence that increased levels of NADPH do really occur is provided by the MTT testing where 0.5 and 5 μM of AM12 unexpectedly led to a moderate but highly significant increase in MTT conversion after a 24-h exposure (Fig. 2a). It has been shown that tetrazolium compounds can be directly reduced by NADPH (Naoi et al. 2010). The moderate but significant decrease of MTT conversion by 0.5 and 5 μM of AM12 after 48 h, however, suggests a diminished viability of the MIN6 cells and conforms to the antioxidative and antiapoptotic function of TRX in insulin-secreting cells (Hotta et al. 1998; Stancill et al. 2019).

This interpretation was supported by the use of the live/dead assay. Even though a concentration-dependent decrease in living cells was visible after 24 h and 48 h, only 10 μM AM12 was sufficient to produce a significant decrease as compared to control. The largely unchanged percentage of dead (ethidium stained) cells was unexpected. Here, fluorescence microscopy provided clarification. When the exposure time was 24 h, neither 0.5 nor 5 μM AM12 affected the total cell number and appearance as well as the number of dead or living cells (see Fig. 4a). After 48-h exposure, 0.5 μM was without effect on cell viability, whereas exposure to 5 μM increased the number of dead cells and decreased the number of living cells. However, the changes in fluorescent labeling were much less impressive than those caused by saponin (see Fig. 4b). 5 μM AM12 also induced a fragmented appearance of the cells, but cells and cell fragments had a high contrast in transmitted light, suggesting that the plasma membrane had remained intact, as is typical for apoptosis (Fadeel 2004).

This conclusion was in principle confirmed by testing the effects on isolated islets. While the cells remained viable during a 24-h exposure to 0.5 and 5.0 μM AM12 in cell culture, most islet cells were labeled by annexin V when the concentration was 5.0 μM. Only a slight difference was apparent between control-cultured islets and islets exposed to 0.5 μM AM12. Here, the annexin labeling was confined to the outer cell layer. Overall, it can be concluded that there is a time window of about 24 h during which AM12 inhibits thioredoxin reductase but has not yet affected viability, even though the onset of apoptosis is underway. Islets appear to be more susceptible than MIN6 cells.

This information was important to check for the functional consequences of thioredoxin inhibition on secretion. Acute exposure of perifused pancreatic islets did not result in a clear-cut effect on insulin secretion, neither at basal nor at stimulatory glucose concentration. Most likely, this lack of acute effect of AM12 is due to a slow cell permeation. Thus, a 24-h exposure during islet culture was used to achieve a sufficient intracellular concentration.

The secretion rate of AM12-exposed islets did not differ from that of control-cultured islets at basal glucose, but both phases of the secretion caused by 30 mM glucose were significantly higher after culture in the presence of 5.0 μM AM12 but not 0.5 μM AM12. Of note, the kinetics was unchanged as was the reversibility to prestimulatory secretion levels during washout. Unexpectedly, the effect on [Ca^2+^]_i_ was virtually the opposite; islets exposed to 5.0 μM AM12 showed a much-reduced increase during glucose stimulation, whereas the increase of islets exposed 0.5 μM AM12 was closely similar to one of control-cultured islets. Part of this paradoxical effect may reflect the predominant representation of the outer islet cell layer in the Fura fluorescence signal. These cells showed a loosened structure (see Fig. 5) and may no longer be connected via connexons, which synchronize the [Ca^2+^]_i_ signals of the islet (Farnsworth and Benninger 2014). Even in view of these methodological aspects it seems that TrxR inhibition by AM12 increases the efficacy of the [Ca^2+^]_i_ signal for exocytosis.

There are several, not mutually exclusive hypotheses to explain this observation: Firstly, TRX has a tonic inhibitory effect on exocytosis, similar to what has been suggested earlier (Ivarsson et al. 2005), which is relieved by the inhibition of TRX reductase. Secondly, the diminished consumption of NADPH, resulting from the inhibition of the TRX-R, enables a higher activity of other NADPH-dependent processes. An NADPH-dependent process which has been suggested to result in the amplification of depolarization-triggered insulin secretion is the de-SUMOylation of proteins involved in exocytosis, such as syntaxin (Ferdaoussi et al. 2015; Davey et al. 2019). Finally, the inhibition of TRX reductase can increase the level of reactive oxygen species, which does not only pave the way to cell death (Matsuzawa 2017) but may also play a signaling role in insulin secretion, as has been suggested for cytosolic H_2_O_2_ (Plecitá-Hlavatá et al. 2020).

To gain further insight into the underlying events and to better separate effects on secretion from effects on viability, it would be desirable to exert a more acute inhibition of TRX-R. Thus, the development of derivates of AM12 with accelerated membrane permeation is warranted. The present observations suggest that research on the deficit of functional beta cell mass in type 2 diabetes will profit from such a pharmacological tool.

## Data Availability

The compound AM12 and the datasets generated during and/or analyzed during the current study are not publicly available but are available from the corresponding author on reasonable request.

## References

[CR1] Andermark V, Göke K, Kokoschka M, Abu El Maaty MA, Lum CT, Zou T, Sun RWY, Aguilo E, Oehninger L, Rodriguez L, Bunjes H, Wölfl S, Che CM, Ott I (2016). Alkynyl gold(I) phosphane complexes: evaluation of structure–activity-relationships for the phosphane ligands, effects on key signaling proteins and preliminary in-vivo studies with a nanoformulated complex. J Inorg Biochem.

[CR2] Attie AD (2015). How do reducing equivalents increase insulin secretion?. J Clin Invest.

[CR3] Berridge MV, Tan AS (1993). Characterization of the cellular reduction of 3-(4,5-dimethylthiazol-2-yl)-2,5-diphenyltetrazolium bromide (MTT): subcellular localization, substrate dependence, and involvement of mitochondrial electron transport in MTT reduction. Arch Biochem Biophys.

[CR4] Berridge MV, Tan AS, McCoy KD, Kansara M, Rudert F (1996). CD95 (Fas/Apo-1)-induced apoptosis results in loss of glucose transporter function. J Immunol.

[CR5] Chen J, Saxena G, Mungrue IN, Lusis AJ, Shalev A (2008). Thioredoxin-interacting protein: a critical link between glucose toxicity and beta-cell apoptosis. Diabetes.

[CR6] Davey JS, Carmichael RE, Craig TJ (2019). Protein SUMOylation regulates insulin secretion at multiple stages. Sci Rep.

[CR7] Fadeel B (2004). Plasma membrane alterations during apoptosis: role in corpse clearance. Antioxid Redox Signal.

[CR8] Farnsworth NL, Benninger RK (2014). New insights into the role of connexins in pancreatic islet function and diabetes. FEBS Lett.

[CR9] Ferdaoussi M, Dai X, Jensen MV, Wang R, Peterson BS, Huang C, Ilkayeva O, Smith N, Miller N, Hajmrle C, Spigelman AF, Wright RC, Plummer G, Suzuki K, Mackay JP, van de Bunt M, Gloyn AL, Ryan TE, Norquay LD, Brosnan MJ, Trimmer JK, Rolph TP, Kibbey RG, Manning Fox JE, Colmers WF, Shirihai OS, Neufer PD, Yeh ET, Newgard CB, MacDonald PE (2015). Isocitrate-to-SENP1 signaling amplifies insulin secretion and rescues dysfunctional β cells. J Clin Invest.

[CR10] Henquin JC (2000). Triggering and amplifying pathways of regulation of insulin secretion by glucose. Diabetes.

[CR11] Holmgren A, Lu J (2010). Thioredoxin and thioredoxin reductase: current research with special reference to human disease. Biochem Biophys Res Commun.

[CR12] Hotta M, Tashiro F, Ikegami H, Niwa H, Ogihara T, Yodoi J, Miyazaki J (1998). Pancreatic beta cell-specific expression of thioredoxin, an antioxidative and antiapoptotic protein, prevents autoimmune and streptozotocin-induced diabetes. J Exp Med.

[CR13] Ivarsson R, Quintens R, Dejonghe S, Tsukamoto K, in’ t Veld P, Renström E, Schuit FC (2005). Redox control of exocytosis: regulatory role of NADPH, thioredoxin, and glutaredoxin. Diabetes.

[CR14] Janjic D, Wollheim CB (1992). Islet cell metabolism is reflected by the MTT (tetrazolium) colorimetric assay. Diabetologia.

[CR15] Lu J, Holmgren A (2014). The thioredoxin antioxidant system. Free Radic Biol Med.

[CR16] Martin SJ, Reutelingsperger CP, McGahon AJ, Rader JA, van Schie RC, LaFace DM, Green DR (1995). Early redistribution of plasma membrane phosphatidylserine is a general feature of apoptosis regardless of the initiating stimulus: inhibition by overexpression of Bcl-2 and Abl. J Exp Med.

[CR17] Matsuzawa A (2017). Thioredoxin and redox signaling: roles of the thioredoxin system in control of cell fate. Arch Biochem Biophys.

[CR18] Meyer A, Bagowski CP, Kokoschka M, Stefanopoulou M, Alborzinia H, Can S, Vlecken DH, Sheldrick WS, Wölfl S, Ott I (2012). On the biological properties of alkinyl phosphine gold(I) complexes. Angew Chem Int Ed Eng.

[CR19] Miyazaki J, Araki K, Yamato E, Ikegami H, Asano T, Shibasaki Y, Oka Y, Yamamura K (1990). Establishment of a pancreatic beta cell line that retains glucose-inducible insulin secretion: special reference to expression of glucose transporter isoforms. Endocrinology.

[CR20] Mosmann T (1983). Rapid colorimetric assay for cellular growth and survival: application to proliferation and cytotoxicity assays. J Immunol Methods.

[CR21] Mustacich D, Powis G (2000). Thioredoxin reductase. Biochem J.

[CR22] Naoi T, Shibuya N, Inoue H, Mita S, Kobayashi S, Watanabe K, Orino K (2010). The effect of tert-butylhydroquinone-induced oxidative stress in MDBK cells using XTT assay: implication of tert-butylhydroquinone-induced NADPH generating enzymes. J Vet Med Sci.

[CR23] Panten U, Rustenbeck I (2008). Fuel-induced amplification of insulin secretion in mouse pancreatic islets exposed to a high sulfonylurea concentration: role of the NADPH/NADP+ ratio. Diabetologia.

[CR24] Papadopoulos NG, Dedoussis GV, Spanakos G, Gritzapis AD, Baxevanis CN, Papamichail M (1994). An improved fluorescence assay for the determination of lymphocyte-mediated cytotoxicity using flow cytometry. J Immunol Methods.

[CR25] Patwari P, Higgins LJ, Chutkow WA, Yoshioka J, Lee RT (2006). The interaction of thioredoxin with Txnip. Evidence for formation of a mixed disulfide by disulfide exchange. J Biol Chem.

[CR26] Plecitá-Hlavatá L, Jabůrek M, Holendová B, Tauber J, Pavluch V, Berková Z, Cahová M, Schröder K, Brandes RP, Siemen D, Ježek P (2020). Glucose-stimulated insulin secretion fundamentally requires H _2_ O _2_ signaling by NADPH oxidase 4. Diabetes.

[CR27] Reinbothe TM, Ivarsson R, Li DQ, Niazi O, Jing X, Zhang E, Stenson L, Bryborn U, Renström E (2009). Glutaredoxin-1 mediates NADPH-dependent stimulation of calcium-dependent insulin secretion. Mol Endocrinol.

[CR28] Stancill JS, Broniowska KA, Oleson BJ, Naatz A, Corbett JA (2019). Pancreatic β-cells detoxify H_2_O_2_ through the peroxiredoxin/thioredoxin antioxidant system. J Biol Chem.

[CR29] Urig S, Becker K (2006). On the potential of thioredoxin reductase inhibitors for cancer therapy. Semin Cancer Biol.

[CR30] Westermark PO, Kotaleski JH, Björklund A, Grill V, Lansner A (2007). A mathematical model of the mitochondrial NADH shuttles and anaplerosis in the pancreatic beta-cell. Am J Physiol Endocrinol Metab.

